# RE: Impact on psychiatrists in intellectual disability of Court of Protection orders for section 49 (Mental Capacity Act) reports: online survey

**DOI:** 10.1192/bjb.2022.71

**Published:** 2023-02

**Authors:** Niraj Singh, Asit Biswas

**Affiliations:** Consultant Psychiatrist in Intellectual Disability, Nottinghamshire Healthcare NHS Trust, and Executive Committee Member, Royal College of Psychiatrists, UK. Email: nirajsingh1@nhs.net; Consultant Psychiatrist in Intellectual Disability, Leicestershire Partnership NHS Trust, and Vice-Chair of the Faculty of Intellectual Disability, Royal College of Psychiatrists, UK

In response to this article in the *BJPsych Bulletin*^1^, we note with great concern the statistics on the impact of section 49 reports on intellectual disability psychiatrists. Of the 104 psychiatrists who responded, 65.4% had been ordered to undertake a section 49 report; 51.5% of those had been asked to provide an opinion outside their subjective expertise, 25% were somewhat or fully confident in writing reports and 85% stated that they experienced stress as a result. This is a huge indictment of the impact that section 49 reports have had on workforce well-being. Psychiatrists with already heavy workloads in stretched services have little recourse or ability to negotiate these instructions. We therefore support and reinforce the proposals put forward by Perera et al.

- Training must be provided to doctors in mental capacity assessment and section 49 report writing.
- A dedicated section 49 report team should be available within trusts. A specialty-specific psychiatrist should be recruited to prepare those reports, which are not limited to intellectual disability psychiatry but also extend to old age psychiatry, neuropsychiatry, brain injury services and adult mental health among others. Where warranted, neuropsychological expertise must be available as part of the team for cognitive testing.
- Where a psychiatrist cannot be recruited to prepare these reports, time must be incorporated into job planning.
- Trusts should continue to survey the impact of this workload.
- Terms of arrangement must be developed by trusts in order to ensure psychiatrists have adequate support and time to prepare section 49 reports.
- Administrative support needs to be provided for the reports both for documentation and communication with the legal team.

## References

[1] Perera S, Leyland N, Coshever J. Impact on psychiatrists in intellectual disability of Court of Protection orders for section 49 (Mental Capacity Act) reports: online survey. BJPsych Bull [Epub ahead of print] 2 Mar 2022. Available from: 10.1192/bjb.2022.10. PMC10214433 35236534

